# FlyMine: an integrated database for *Drosophila *and *Anopheles *genomics

**DOI:** 10.1186/gb-2007-8-7-r129

**Published:** 2007-07-05

**Authors:** Rachel Lyne, Richard Smith, Kim Rutherford, Matthew Wakeling, Andrew Varley, Francois Guillier, Hilde Janssens, Wenyan Ji, Peter Mclaren, Philip North, Debashis Rana, Tom Riley, Julie Sullivan, Xavier Watkins, Mark Woodbridge, Kathryn Lilley, Steve Russell, Michael Ashburner, Kenji Mizuguchi, Gos Micklem

**Affiliations:** 1 Department of Genetics, University of Cambridge, Cambridge, CB2 3EH, UK; 2 Department of Biochemistry, University of Cambridge, Cambridge, CB2 1GA, UK; 3 Cambridge Computational Biology Institute, Department of Applied Mathematics and Theoretical Physics, University of Cambridge, Cambridge, CB3 OWA, UK; 4 National Institute of Biomedical Innovation 7-6-8 Saito-Asagi, Ibaraki, Osaka 567-0085, Japan

## Abstract

FlyMine is a data warehouse that addresses one of the important challenges of modern biology: how to integrate and make use of the diversity and volume of current biological data. Its main focus is genomic and proteomics data for *Drosophila *and other insects. It provides web access to integrated data at a number of different levels, from simple browsing to construction of complex queries, which can be executed on either single items or lists.

## Abstract 2

This novel web-based database provides unique accessibility and querying of integrated genomic and proteomic data for *Drosophila *and *Anopheles*.

## Rationale

With the completion of increasing numbers of genome sequences has come an explosion in the development of both computational and experimental techniques for deciphering the functions of genes, molecules and their interactions. These include theoretical methods for deducing function, such as analysis of protein homologies, structural domain predictions, phylogenetic profiling and analysis of protein domain fusions, as well as experimental techniques, such as microarray-based gene expression and transcription factor binding studies, two-hybrid protein-protein interaction screens, and large-scale RNA interference (RNAi) screens. The result is a huge amount of information and a current challenge is to extract meaningful knowledge and patterns of biological significance that can lead to new experimentally testable hypotheses. Many of these broad datasets, however, are noisy and the data quality can vary significantly. While in some circumstances the data from each of these techniques are useful in their own right, the ability to combine data from different sources facilitates interpretation and potentially allows stronger inferences to be made. Currently, biological data are stored in a wide variety of formats in numerous different places, making their combined analysis difficult: when information from several different databases is required, the assembly of data into a format suitable for querying is a challenge in itself. Sophisticated analysis of diverse data requires that they are available in a form that allows questions to be asked across them and that tools for constructing the questions are available. The development of systems for the integration and combined analysis of diverse data remains a priority in bioinformatics. Avoiding the need to understand and reformat many different data sources is a major benefit for end users of a centralized data access system.

A number of studies have illustrated the power of integrating data for cross-validation, functional annotation and generating testable hypotheses (reviewed in [1,2]). These studies have covered a range of data types; some looking at the overlap between two different data sets, for example, protein interaction and expression data [3-6] or protein interaction and RNAi screening results [7], and some combining the information from several different types of data [8-12]. Studies with *Saccharomyces cerevisiae*, for example, have indicated that combining protein-protein interaction and gene expression data to identify potential interacting proteins that are also co-expressed is a powerful way to cross-validate noisy protein interaction data [3-6]. A recent analysis integrated protein interactions, protein domain models, gene expression data and functional annotations to predict nearly 40,000 protein-protein interactions in humans [9]. In addition, combining multiple data sets of the same type from several organisms not only expands coverage to a larger section of genomes of interest, but can help to verify inferences or develop new hypotheses about particular 'events' in another organism. Alternatively, finding the intersection between different data sets of the same type can help identify a subset of higher-confidence data [2]. In addition to examination of different data sources within an organism, predicted orthologues and paralogues allow cross-validation of datasets between different organisms. For example, identification of so-called interologues (putative interacting protein pairs whose orthologues in another organism also apparently interact), can add confidence to interactions [13].

Biological data integration is a difficult task and a number of different solutions to the problem have been proposed (for example, see [14,15] for reviews). A number of projects have already tackled the task of data integration and querying, and the methods used by these different systems differ greatly in their aims and scope (for a review of the different types of systems, see [15]). Some, for example, do not integrate the data themselves but perform fast, indexed keyword searches over flat files. An example of such a system is SRS [16]. Other systems send queries out to several different sources and use a mediated middle layer to integrate the information (so called mediated systems such as TAMBIS [17], DiscoveryLink [18] and BioMoby [19]). Although these systems can provide a great range of data and computational resources, they are sensitive to network problems and data format changes. In addition, such systems run into performance issues when running complex queries over large result sets. Finally, like FlyMine, some systems integrate all the data into one place - a data warehouse (for example, GUS [20], BioMart [21], Biozon [22], BioWarehouse [23], GIMS [24], Atlas [25] and Ondex [26]). Our objective was to make a freely available system built on a standard platform using a normal schema but still allowing warehouse performance. This resulted in the development of InterMine [27], a generic system that underpins FlyMine. A particular feature of InterMine is the way it makes use of precomputed tables to enhance performance. Another key component is the use of ontologies that provide a standardized system for naming biological entities and their relationships and this aspect is based on the approach taken by the Chado schema [28]. For example, a large part of the FlyMine data model is based on the Sequence Ontology (a controlled-vocabulary for describing biological sequences) [29]. This underlying architecture is discussed in more detail under 'System architecture'.

Another objective for FlyMine was to provide access to the data for bioinformatics experts as well as bench biologists with limited database (or bioinformatics) knowledge. FlyMine provides three kinds of web-based access. First, the Query Builder provides the most advanced access, allowing the user to construct their own complex queries. Second, a library of 'templates' provides a simple form-filling interface to predefined queries that can perform simple or complex actions. It is very straightforward to convert a query constructed in the Query Builder into a template for future use. Finally, a Quick Search facility allows users to browse the data available for any particular item in the database and, from there, to explore related items. This level of query flexibility combined with a large integrated database provides a powerful tool for researchers.

Below we briefly outline the data sources available in the current release of FlyMine and provide details of how these data can be accessed and queried. This is followed by examples illustrating some of the uses of FlyMine and the advantage of having data integrated into one database. Finally, we describe our future plans, and how to get further help and information.

## Data

The aim of FlyMine is to include large-scale functional genomic and proteomic data sets for a range of model organisms, with the main focus currently being on *Drosophila *and *Anopheles *species. So far we have loaded a wide range of data and these are summarized in Table 1.

**Table 1:** Summary of data sources available in release 6.0 of FlyMine

Data	Organism	Source	Reference
Genome annotation	*D. melanogaster*, *A. gambiae*, *D. pseudoobscura*, *A. mellifera*	FlyBase Ensembl	[50,51] [39,40]
Protein annotation	*D. melanogaster*, *D. pseudoobscura*, *A. gambiae*, *A. mellifera*, *C. elegans*, *S. cerevisiae*	UniProtKB version 8.9	[35,36]
Protein family and domain annotation	*D. melanogaster*, *A. gambiae*, *C. elegans*	InterPro version 12.0	[41,42]
Protein-protein interactions	*D. melanogaster*, *C. elegans*, *S. cerevisiae*	IntAct	[65-74]*
RNAi phenotypes	*C. elegans* *D. melanogaster*	WormBase *Drosophila *RNAi screening center	[75-78] [79-85]
Three-dimensional structural domain predictions	*D. melanogaster*	Kenji Mizuguchi	Personal communication
MicroArray GeneExpression	*D. melanogaster*	ArrayExpress FlyAtlas	[86] [52]
Orthologues/paralogues	*D. melanogaster*, *A. gambiae*, *C. elegans*, *A. mellifera*, *D. pseudoobscura *+ others (see [64])	InParanoid	[45,46]
GO annotation and the Gene Ontology	*D. melanogaster*, *C. elegans*, *A. gambiae *+ others (see [64])	Gene Ontology site UniProtKB GOA	[87,88] [35,36]
DNAse1 footprints.	*D. melanogaster*	flyreg version 2.0	[89,90]
Transcriptional *cis*-regulatory modules	*D. melanogaster*	REDfly	[91]
Whole genome tiling path	*D. melanogaster*	Cambridge University	
INDAC microarray oligo set	*D. melanogaster*	INDAC Version 1.0	[92]
P-element insertions and deletions	*D. melanogaster*	DrosDel	[43,44]
Homophila	Human disease to *D. melanogaster*	Homophila version 2.1	[93,94]

*In addition, a number of protein interactions and complexes from smaller scale experiments are available (see the Protein Interaction aspect on the FlyMine web site for details).

Currently, we can load any data that conform to several different formats: GFF3 [30] for genome annotation and genomic features (for example, Dnase I footprints, microarray oligonucleotide and genome tiling amplimers), PSI-MI [31,32] for data describing protein interactions or complexes, MAGE [33,34] for microarray expression data, XML files from the UniProt Knowledgebase (UniProtKB) [35,36] and the OBO flat file format describing the Gene Ontology (GO) [37] and gene association files for GO annotations [38]. In addition, we can also import data from the Ensembl [39,40], InterPro [41,42] and DrosDel [43,44] database schemas to the FlyMine data model, enabling data from these databases to be loaded and updated regularly. Several smaller-scale data sources that currently do not conform to any standard have also been incorporated, such as RNAi data, orthologue data generated by InParanoid [45,46] and three-dimensional protein structural domain predictions (K Mizuguchi, unpublished).

When building FlyMine, data are parsed from source formats and loaded into a central database. Queries are executed on this database with no need to access the original source data. Overlapping data sets are integrated by common identifiers, for example, genes from different sources may be merged by identifier or symbol. FlyMine is rebuilt with updated versions of source data about every three months. Table 2 summarizes the current number of objects in some of the main FlyMine classes.

**Table 2:** The number of unique occurences of some of the main types ofobjects in FlyMine release 6.0

Object type	Number of unique objects in FlyMine
Gene	225,059
Protein	79,640
Protein interactions	45,262
Protein structures	11,363
Regulatory regions	2,361
Orthologues	275, 062
RNAi screens	146
Microarray results	1,517,787

### Aspects

**Figure 1:**
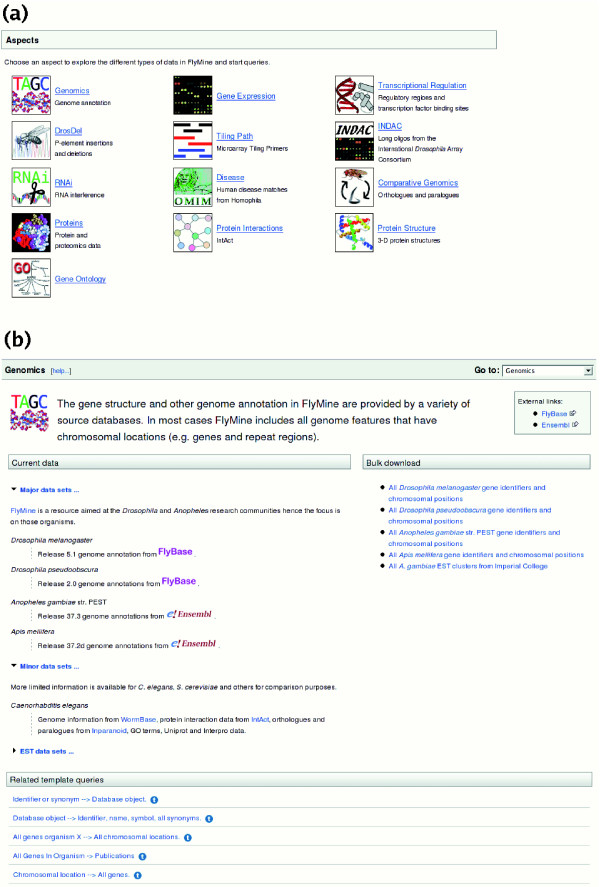
Aspects. FlyMine groups data into 'aspects', each of which provide a 'homepage' for a different category of data. **(a) **The aspects available in FlyMine release 6.0. Each aspect page can be accessed by clicking on its icon or title. **(b) **Example aspect page: Genomics Aspect. Each aspect provides background information on the origin of each of its source datasets through a short description, and references if available. Likewise, links are provided to any source databases. Convenient bulk datasets are made available for browsing, or export in standard format. In addition, relevant template queries and classes for use as starting points in the query builder are provided.

As one starting point, the FlyMine website presents the principle data types grouped together as different 'aspects' (Figure 1a), such as protein interactions or gene expression. Each aspect provides background information on the origin of particular source datasets, including literature references and links to source databases, access to convenient derivative bulk datasets for browsing or export in standard format, as well as pre-defined template queries and classes for use as starting points in the query builder (Figure 1b). The template queries available for a particular aspect range from simple queries to retrieve just one data type to more complex queries that incorporate data from other aspects. Thus, aspects allow researchers to easily focus on a particular type of data, while still being able to query multiple data types at once and, for instance, easily identify relevant template queries.

## User interfaces

**Figure 2:**
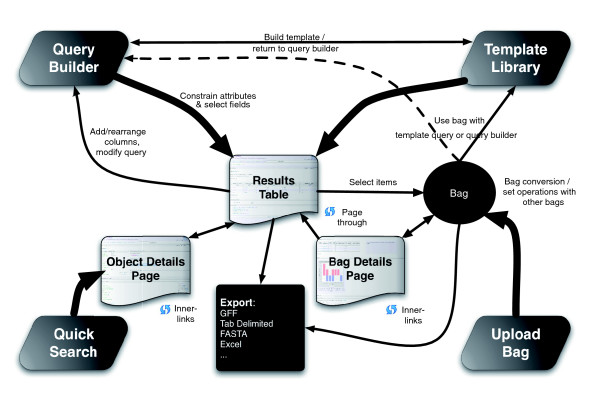
Map of the user interface. The icons at the four corners represent entry points allowing (clockwise from top left): the construction of new queries; search for/running of pre-made 'template' queries; uploading of lists of objects (which can be used in queries); or a quick search for objects based on their identifiers or synonyms. Running a query produces a results page, allowing browsing and paging through results and selection of items for inclusion in lists or export in a variety of formats. Lists can be saved between sessions, set operations can be carried out on multiple lists and the list details page summarizes list contents and provides other tools that operate on lists.

FlyMine allows access to the integrated data in multiple ways, as illustrated by Figure 2. There are four entry points into FlyMine, the Quick Search, Template Queries, the Query Builder and Lists. These entry points can take the user to an object details page (from Quick Search), a results page (from the Query Builder or a Template Query), or a list details page. Each of these is described in more detail below.

### Object details pages

**Figure 3:**
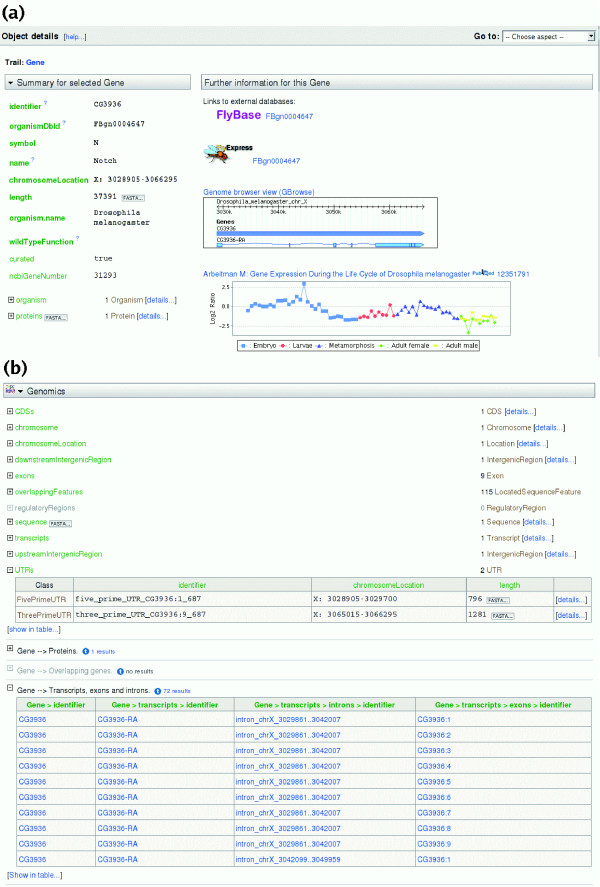
The object details page for the *D. melanogaster Notch *gene. **(a) **The left-hand side of the top pane provides a summary for the object being viewed, including the main describing attributes. To the right of the summary, a 'further information' section provides link-outs to relevant databases and access to relevant graphical tools, in this case GBrowse and a graph showing expression of the *Notch *gene during the *D. melanogaster *life cycle. **(b) **For easy navigation, further information for the object being viewed is divided into different sections according to the different 'aspects'. For each aspect, related objects (for example, proteins, for a gene page) from referenced classes and the results of relevant template queries (run using the object being viewed as the variable) are available in-line. From each related object a link is provided to the object details page for that object via the [details...] links. In this example, the objects referenced in the overlappingFeatures class are shown and the results of one template query have been expanded.

Each entry in the database, such as a gene or protein, is known as an 'object'. Every object can be viewed through an 'object details' page, from which all information relating to that object can be accessed. The page first provides a summary of the object, where the main attributes are displayed. For example, for a gene, the summary includes attributes for gene names, symbols and identifiers, chromosome and genome location, a link to the available protein information and a link to additional attributes (Figure 3a). For sequence features (for example, transcripts, exons, genes) it is possible to retrieve FASTA-formatted data from the object details page. Where applicable, object details pages include links to embedded graphical viewing tools: for example, GBrowse [47,48] for sequence features and Jmol [49] for protein structures. In addition, where applicable, links to external databases are provided. For example, each *Drosophila melanogaster *gene object details page in FlyMine has a link to the gene report page in FlyBase [50,51] (Figure 3a) and *vice versa*. In addition to the summary, each object details page provides further information available for the object, divided into subsections corresponding to the different 'aspects' for easy navigation (Figure 3b). Each aspect provides links to related objects. These can be expanded to show a summary of its attributes, together with a link to its own object details page. Much of the information in the database can be accessed by following the links between related objects. For example, accessing the 'details' link for a referenced protein object shows the object details page for that protein, which, in turn, provides other information, including family, domain, predicted structure and interaction data, where available. In addition, each aspect section provides a set of relevant template queries for the type of object being viewed that are executed when the page is loaded. The number of results that will be returned by a template is displayed, and where there are none the line is grayed out. A useful feature is that the results for each template query can be viewed in-line on the object details page by selecting the adjacent '+' symbol, thus increasing the amount of additional information about an object quickly and easily (Figure 3b)

### Quick search

The quick search option provides the simplest way to access FlyMine by allowing users to search all identifiers and synonyms at once (using wild cards if necessary). This takes the user either directly to an object details page (if the search returns just one object) or to a list of objects that match the query, together with links to the corresponding details pages for each object. The quick search option therefore provides a simple way for users to retrieve objects where a name or identifier is known, and to browse all data available for that object and related objects.

### Template queries

**Figure 4:**
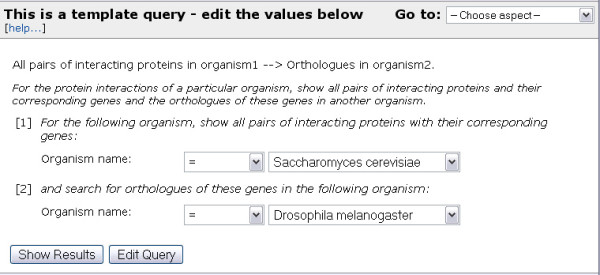
Example of a template query. The template 'All pairs of interacting proteins in organism1 → Orthologues in organism2' is shown. All template queries have a 'short name' and a longer description. The short name aids visual scanning of available templates. Template queries consist of a form, in which the user is able to adjust the default constraints on classes and attributes. Each box, or variable, can be constrained to either a single value or, if appropriate, to a list of objects (if the user has lists of the appropriate type stored in their history). The buttons 'Show Results' and 'Edit Query' provide the user with the choice of either viewing the results page, or viewing the query in the query builder, where it may be modified.

To enable users to quickly and easily carry out a range of queries, both simple and complex, FlyMine includes a growing library of predefined 'template' queries. Each template provides a simplified view of an underlying query by means of a text description and one or more editable constraints (Figure 4). Default values are always provided for the constraint fields, to illustrate the type of values required and so that example output can be generated immediately. The constraints are filters on one or more types of data, for example, by restricting the search to one particular organism, and can either be a single value or a list of objects. The ability to constrain a template query to a list of objects is a particularly powerful feature, as, for instance, it allows a query to be run on thousands of genes in one operation. Templates range from relatively simple queries, such as 'Search for genes annotated with a particular GO term' or 'Find the predicted orthologues for a particular gene' to more complex queries, integrating two or more datasets: for example, 'for the protein product of a particular gene, show any proteins it has been shown to interact with and for these proteins their Pfam domain content'. To aid navigation of template queries, a keyword search facility is available, which grades results by similarity to the search terms. In addition, users are able to select individual templates and add them to a 'favorites' list available from their 'MyMine' account (see below). Importantly, since template queries can be returned to the query builder, it is possible for a user to modify any template. For instance, constraints can be removed or further ones added, or the choice of output columns can be changed. Thus, templates can be used as a starting point in the generation of related queries. This feature also acts as a training tool for the query builder, by allowing users to first understand the function of a query through executing it, and then to examine the underlying structure of the query in the query builder.

New templates can be added at any time: both users and the FlyMine team can derive templates from queries using the web interface, although currently only those from the team are visible to all users. Creation of a template from a query is carried out by completing a short web form in which constraint fields are chosen and labeled, and the template is described. Making and saving templates complements saving queries (see 'Personal FlyMine accounts: MyMine' below) and allows users to build their own library of useful functionality. Templates can be exported and imported as XML, thus facilitating the sharing of templates between users.

### The query builder

The query builder is a tool that allows users to navigate the FlyMine data model, choosing what columns of data should be output and applying constraints that will limit the output to that of interest. Internally, the FlyMine database is built on top of an object-based data model that defines the way different types of data are related to each other. This model is made up of classes, each of which describes a particular object-type and its relationship to other objects. Data with similar properties are grouped together into the same class. For example, there are classes to describe sequence features such as genes and exons. Each class has a set of attributes that define the different types of information that can be recorded in each object of that class. In the case of the Gene class, attributes include fields for name, symbol and identifier among others. Each object in the database is a member of a class; for example, the *zen *gene is an object of the Gene class. The classes are linked together by references that define the relationships between objects in different classes. For example, the Gene class has a reference to the transcript class, which allows a particular gene object to have references to multiple transcript objects.

**Figure 5:**
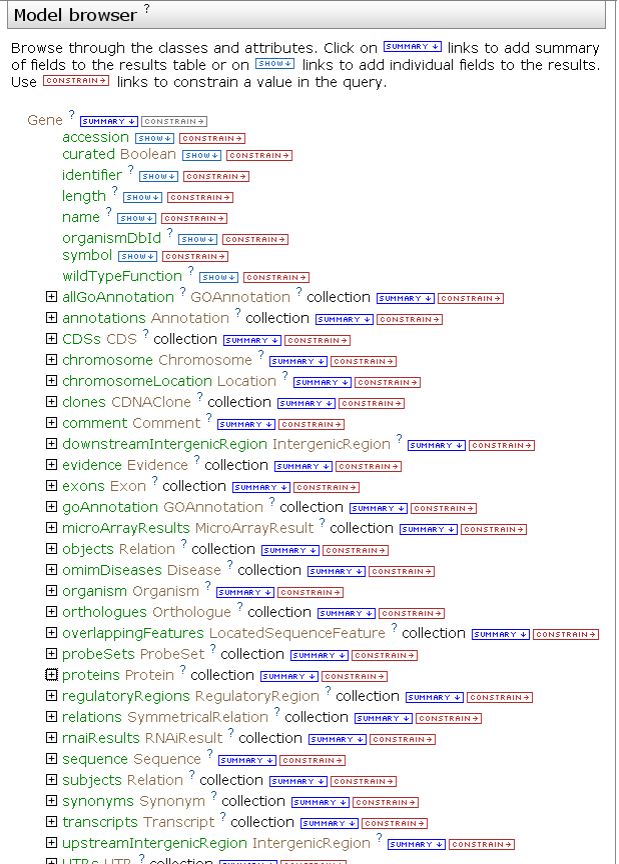
The model browser: part of the FlyMine query builder. The model browser initially displays a user-selected class, from where it is possible to browse to other classes via the references between them. This figure shows the Gene class with some of its attributes and referenced classes. These referenced classes (for example, Organism, Proteins) also appear as attributes of the Gene class.

**Figure 6:**
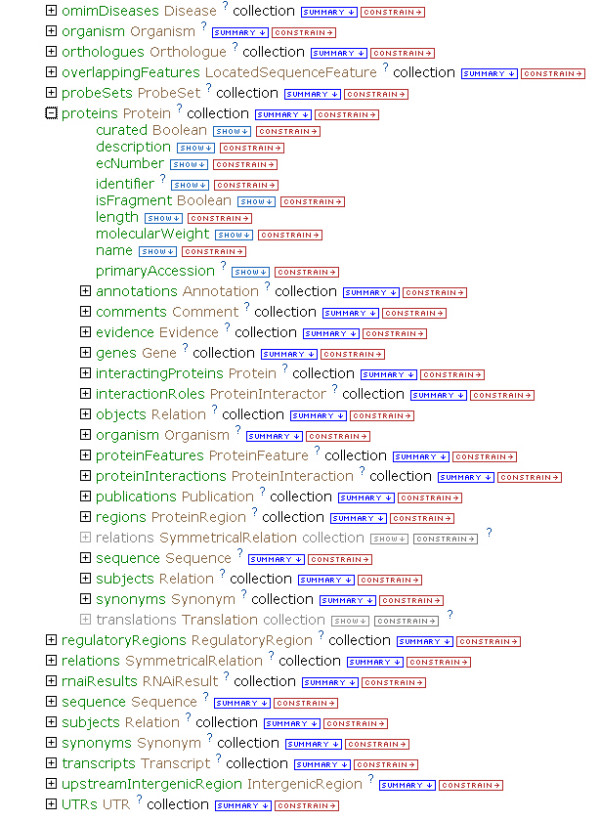
Browsing the data model. Expanding a referenced class in the model browser, in this case the Protein class, shows its attributes and referenced classes, which in turn can also be expanded.

The query builder provides a representation of the data model called the 'model browser' (Figure 5). The model browser allows users to navigate through the data model via the references described above. Since the references from one class to another are also attributes of each class (for example, the Transcript class is an attribute of the Gene class), this provides a natural way to build complex queries as one can seamlessly navigate between classes (Figure 6). Since the model is built largely from standards and ontologies that model biological concepts, the paths that can be taken through the model logically reflect the underlying biology: for example, genes are linked to transcripts and to proteins that are linked to protein interaction data. Therefore, it is possible to construct queries without specific knowledge of the data model and without using a query programming language.

**Figure 7:**
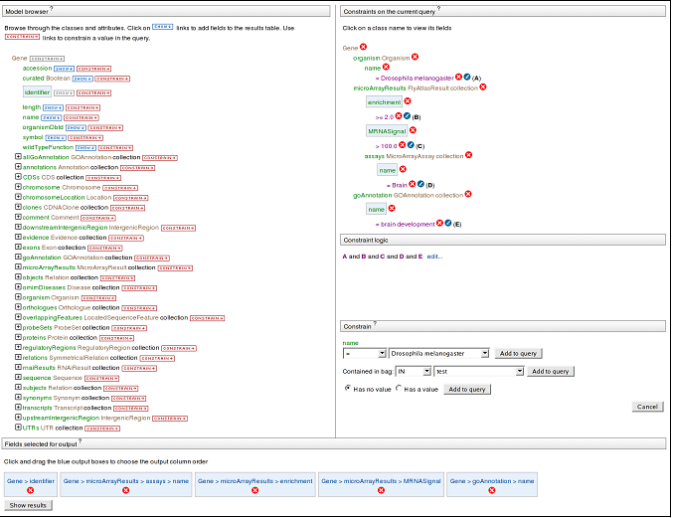
The FlyMine query builder. The query builder consists of three sections that show the data model, constraints and output columns. The model browser in the top left displays the starting class and its attributes (in this case the Gene class and its attributes, including references to other classes). Next to each attribute in the model browser are 'show' and 'constrain' buttons. Clicking on the constrain button brings up a box on the right hand side where a constraint can be specified. The screenshot shows the constraint box that appears after clicking on 'constrain' next to the organism name attribute. Any constraints added are listed in the constraints list on the top right. In the example shown, constraints have already been added to the organism name, attributes for microarray results and assay and for GO annotation name. The query therefore combines four different types of data. Clicking on a 'show' button adds that attribute to the 'Fields selected for output' pane at the bottom of the query builder. In this example the attributes for gene name, microarray assay, mRNA fold-enrichment, mRNA signal and GO annotation name have been added. Any attributes selected in this way will appear in the query results. The 'Show results' button runs the query.

A query can be thought of as a series of filters (constraints) applied to classes and their attributes. Constraints are used to extract an interesting subset of the total data. As a trivial example, by selecting the Gene class and applying the constraint 'symbol= *zen*', just the *zen *Gene object is returned. Thus, queries can be built by selecting sets of classes that contain the data of interest, and applying constraints to define the subset of data required. The references between the classes mean that although one starts building a query from a single class, constraints can easily be added to related classes and their attributes. To add a constraint, the user selects the 'constrain' button next to the chosen class or attribute. This brings up a form allowing the constraint to be defined. Details of constraints added appear on the right-hand pane of the query builder (Figure 7). If more than one constraint is added, each constraint becomes labeled sequentially (A, B, C... and so on). By default different constraints are combined with the logical AND operator but this can be edited to use OR and brackets can be used to define operator order.

Importantly, users are also able to configure the results table they would like to view (Figure 7). In the same way that any attribute can be constrained, any attribute can also be added as an output column. Each attribute in the model browser has a 'show' button that can be used to add it to a 'Fields selected for output' list underneath the model browser. Such columns selected for the results table can be removed or their order changed either in the query builder or later, once the results table has been created.

The query builder has the advantage that users are not confined to filling in pre-defined forms that potentially restrict the diversity and complexity of queries that can be constructed. Although this kind of form-filling functionality is in fact provided by template queries, it is always possible to return to the query building page to modify a template. We expect that it will take more effort to learn to use the query builder compared to using template queries, but the reward is greater flexibility.

### Lists

Often, it is useful to be able to run queries and perform other operations on lists of objects. In addition, the ability to save sets of data from one query for use in another allows complex queries to be built up in stages. In order to enable researchers to constrain their queries to a set of items (which can be any object in the database with a name or identifier), FlyMine includes a facility to collect items into lists. Lists can be generated by selecting columns (or members of columns) from query results, or by loading a list of identifiers directly (by typing, copying and pasting or by file upload). Importantly, lists can be used to constrain both template queries and queries created using the query builder. For example, for a particular list of interesting genes it is possible to run a template query that will return all their GO annotations. In addition, it is possible to perform set operations to combine lists of data in different ways. Currently, it is possible to subtract the contents of lists, or find their union or intersection. Lists of data can be stored in the user's MyMine account (see below) for future use or reference. Some of the applications of lists are described in more detail in the Discussion.

The list upload facility matches the contents of an entered list with objects in the database. Users are asked to select the 'type' of list they wish to create (for example, a 'Gene' list or a 'Protein' list). Items in the list that do not match the 'type' of list to be created, that are found only as synonyms, that are duplicated in the database (for example, the same identifier from two different organisms), or that do not exist at all in the database are reported. Then, before the list is created, the user has the option to add or discard the above non-standard matches. This facility should aid users in producing lists containing the correct objects and will be useful in its own right for checking the contents of large lists.

List details pages provide further information on the set of objects in a particular list and also run appropriate template queries automatically on the list contents. List details pages are being further developed to provide additional viewing and analysis tools (see 'Future directions'). For example, for a list details page for gene objects, the page currently provides a graphical summary of the expression of the gene set (currently according to the FlyAtlas data [52]). Also, from such a graph it is possible to create additional lists, containing subsets of the data represented by the different graph columns.

### Viewing and analysis of results

An essential component of the web interface is the ability to view and further analyze the data generated by a query. Results generated from the query builder or from template queries can be browsed (via the object details pages) or exported, thus allowing for well-defined hypothesis-driven analysis as well as more exploratory data analysis. Results can be exported in either tab or comma-delimited formats, uploaded directly into Open Office [53] or Microsoft Excel, or in the case of objects that have sequences, such as genes and proteins, annotation can be exported in GFF3 format [30] or sequences as FASTA files. We plan to increase the choice of export formats in the future.

In addition to browsing object details pages or result tables and exporting these from the database, FlyMine aims to provide tools for viewing and further analysis of results. The availability of such tools, and the ability to seamlessly upload query results to them, will greatly reduce the time and effort required to find suitable analysis software and re-format data for use with them. So far the GBrowse genome browser [47,48] has been integrated into the object details pages, allowing users to view a sequence feature (for example, a gene) of interest in the context of its surrounding region and other features. We have also integrated the Jmol three-dimensional structural viewer [49], enabling users to view the three-dimensional structural domain predictions generated as part of the FlyMine project. To aid viewing of results, a graph of interacting proteins is available on the protein 'object details' page and graphs showing the expression of genes during the *D. melanogaster *life cycle (from [54]) are available on the relevant gene object details pages. Additional analysis tools will be added as FlyMine develops (see 'Future directions') and we also intend to increase the range of external data analysis and visualization packages for which one can directly export data.

### Personal FlyMine accounts: MyMine

By creating a log-in, users are able to activate a personal FlyMine account called MyMine. MyMine allows users to permanently save lists, queries and templates and mark 'favorite templates'. Saved queries and templates can be run directly, edited or exported (as XML) from a MyMine account. Every query executed (whether a template or from the query builder) is automatically saved to the user's 'query history' with a default name, which can be changed. By default, such queries are maintained only for the duration of a particular session, but can be saved permanently to the user's MyMine account when the user is logged in. Users can also save queries directly to their MyMine account from the query builder. To generate a MyMine account users need to provide only their e-mail address and a password. Finally, queries can be exported and imported as XML, thus providing an alternative mechanism for saving queries between sessions or for sharing queries.

## System architecture

FlyMine is built using InterMine [27], which was developed as an integral part of the FlyMine project. InterMine is an open source generic system that allows a query-optimized data warehouse and web interface to be quickly built for any data model. The InterMine code is available for download from the InterMine website [27] under the Lesser General Public License [55]. InterMine will be described in more detail elsewhere and a general overview is provided below.

**Figure 8:**
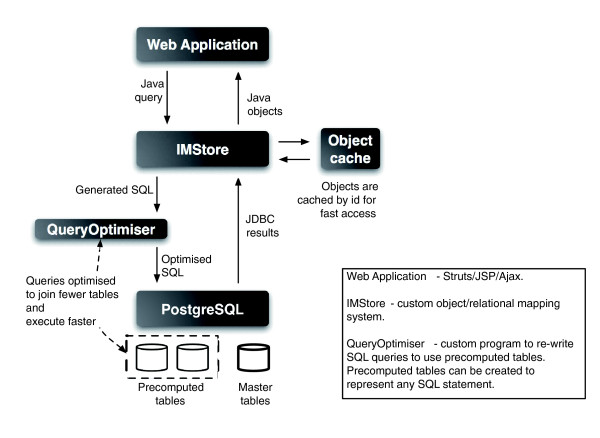
An overview of the InterMine architecture. FlyMine is built using InterMine, which provides the core query system, data integration and web application. The IMStore forms the core of the system; this is accessed from the web application and sends queries to a PostgreSQL relational database. Before execution, the QueryOptimiser re-writes SQL queries to make use of pre-computed tables.

The basic architecture of an InterMine system is shown in Figure 8. At the core of InterMine is IMStore, a custom object/relational mapping system written in Java and optimized for supporting read-only databases. This is accessed from a Struts [56]/JSP/Ajax web application and executes queries in a PostgreSQL [57] relational database. IMStore accepts object queries from a client (the web application), generates SQL to execute in the underlying database and materializes objects from the results. Before final execution, SQL queries are passed to the QueryOptimiser (a Java program developed to enhance InterMine performance), which is able to re-write any SQL query to make use of precomputed tables (analogous to materialized views). The whole query or parts of it may be replaced by one or more precomputed tables; estimates of execution time from the PostgreSQL database are used to decide which possible re-written query will be fastest. This replicates behavior of many commercial database systems but our aim was to use only open source software. Precomputed tables can be created while the database system is running in production so performance can be adapted to match actual usage. This approach separates the definition of the data model from performance optimization, making it possible to tailor the performance of an InterMine warehouse for types of queries not known at design time.

A data model is defined at the object level by an XML file. Java objects, the relational database schema and all model-specific parts of the web application are generated automatically, reducing the maintenance overhead when data model changes are required. Data are loaded as Java objects or XML that conforms to the specified model. Integration of data from multiple sources is configured to define how equivalent objects from different sources should be merged. As different data sources may provide different fields, multiple 'keys' can be defined for a particular type. For example, Gene objects may be merged according to an 'identifier' field or a 'symbol' field. A priority configuration system is used to resolve conflicts between data sources.

InterMine can operate on any data model but we provide an extension specifically for handling biological data, InterMine.bio. This includes a core data model (see below) and a series of 'sources' that include Java code to parse data from a particular data format; for example, UniProtKB, protein interactions and GO annotations each have their own source. The use of data represented by a particular standard facilitates the incorporation of future data into the database. For example, protein interaction data can be represented by the PSI-MI standard [31,32] and by supporting this standard in InterMine we can easily accommodate future data published in this format. Data can also be loaded from an InterMine XML format, allowing the parsing code to be written in a language other than Java. Each source can add classes and fields to extend the data model if required (for example, the protein interaction source adds a ProteinInteraction class) and defines how the data should be integrated. Construction of an InterMine.bio data warehouse (for example, FlyMine) means configuring which sources should be included and specifying the particular organisms or data files to include. This system reduces the development required to update FlyMine and add new data types. It also makes possible the construction of comparable data warehouses for different organisms and data sets.

### The data model

The increasing wealth of data from high-volume biological techniques has driven the development of tools for standardizing the representation of these data to facilitate data comparison, exchange and verification. Huge efforts are currently being put into creating ontologies and other standards to describe different aspects of the data. FlyMine makes use of the Sequence Ontology [29] to define a large part of the data model. This ensures that the relationships between sequence features in the model are biologically meaningful. The Sequence Ontology forms the core of the FlyMine data model, with each term in the ontology becoming a class in the FlyMine data model. A number of additional FlyMine classes allow storing of data, including evidence (the source of the data and publications), experimental and computational results and relationships between objects (for example, orthologues). For data that does not 'fit' to the Sequence Ontology, for example, protein interaction data, additional classes can easily be added to the model as appropriate. The FlyMine data model is designed to evolve as new data types need to be represented.

### Data sources

The data sources in FlyMine come from several public-domain databases and sources (Table 1).

## Discussion

Data integration allows connections to be made between otherwise disparate data sources. In FlyMine it is possible to navigate a path between all types of data in the database and thus combine them in different ways. For example, someone primarily interested in protein-based data, such as interactions or structural data, can easily combine these data with gene-based data, such as GO terms. One of the template queries facilitates such an approach, allowing users to find protein interactions for proteins encoded by genes annotated with a particular GO term. Someone interested in certain proteins that are predicted to interact can navigate to the corresponding information on protein domains/families and any structural information that may be available for these domains. In turn, these data can then be related to further gene-based data, such as GO terms annotated to the genes encoding the proteins or human disease-associated homologues. Orthologues allow further extension of such analysis to data from other organisms, in particular, projection of data from data-rich organisms to those that have had their genomes sequenced but are otherwise less studied. As a simple example, GO annotation can be projected to an organism such as *D. pseudoobscura *for which GO annotations are not yet available: for example, for the *D. pseudoobscura *gene FBgn0080992, GO annotations from the orthologous genes in *D. melanogaster*, *Caenorhabditis elegans*, *Mus musculus *and *S. cerevisiae *can be reported. A template query available from the object details page of this gene ('Show GO terms applied to the orthologues of a particular gene') means that this information is very quickly and easily accessible. Below we describe three more examples that illustrate the use of FlyMine: the first describes the use of overlapping genomic features, the second looks at the identification of interologues and finally some applications of 'lists' are described.

### Overlapping genomic features

Many types of genome data involve the mapping or identification of features in a genome sequence - for example, transcription factor binding sites or transposable element insertion sites. To make such data useful it is often important to know what else has been mapped to, or identified in, that genomic region. Many of the genome features in FlyMine have a reference to a set of overlapping objects, enabling a user to easily retrieve and view anything that overlaps the feature of interest. For any particular chromosome region (for example, a user-defined region, DrosDel deletion or a gene) it is possible to query for features that are mapped to or overlap the region (for example, genes, transcripts, transcription factor binding sites and microarray probes). This allows, for example, the identification of resources that may be available for a particular gene or transcript; for example, *P*-element insertion sites and any resulting deletions from the DrosDel project [43,44] or microarray tiling path amplicons. Such queries, starting from either a chromosome location or a DrosDel deletion, are available as template queries and, for instance, were used by the FlyChip Microarray Facility [58] to identify microarray probes falling within a set of DrosDel deletions, for testing array comparative genomic hybridization (CGH) protocols. Similarly, a template query that returns transcription factor binding sites mapped to a particular genomic region is available. This template also returns the factor that binds to the site (if known) and the gene(s) associated with the site. Representation of these data in GBrowse facilitates visual analysis of such overlapping features. In the future it will also be possible to query for objects that lie within a certain distance upstream or downstream of a particular feature and to distinguish different types of overlap (enclosing, enclosed by, identical, overlapping).

### Identification of interologues

An example of a more complex query is the identification of interologues [13] (for example, proteins in *D. melanogaster *that potentially interact, whose orthologues in *C. elegans *also potentially interact). Since high-throughput two-hybrid protein interaction datasets are prone to a high false positive rate, the identification of interologues leads to increased confidence in particular interactions. For such a query one needs to be able to specify that the orthologues of two proteins that interact also interact with each other. Such a query can be constructed in the FlyMine query builder and is also available as a template query.

In addition to enhancing confidence in interaction data through identification of interologues, potential interactions can be transferred to another organism via orthologue mappings. Such transfer of information has, in previous studies, been used to provide detailed potential interaction networks for a number of organisms, for example, human [59], *S. cerevisiae *to *C. elegans *[13] and between *S. cerevisiae*, *D. melanogaster*, *C. elegans *and *Helicobacter pylori *[60]. The inclusion in FlyMine of interaction datasets from a number of model organisms (currently *D. melanogaster*, *C. elegans *and *S. cerevisiae*), together with the orthologue predictions between these organisms and other organisms for which large-scale interaction datasets are not yet available, allows FlyMine to be used to infer interactions in organisms without their own protein interaction datasets.

### The use of 'lists'

The ability to save lists of objects provides additional power, enabling queries to operate on a particular set of user defined data. Since many large scale experiments, such as microarray studies, produce large sets of potentially interesting genes that need to be investigated further, the ability to confine queries to such a set immediately provides researchers with a tool to investigate those genes without having to look each one up in several different databases. In addition, lists allow logical operations such as unions, intersections and subtraction to be performed. For instance, if one wishes to identify all the *Anopheles gambiae *genes that do not have a predicted orthologue in *D. melanogaster*, one could create a list of *Drosophila *genes with orthologues in *A. gambiae*, a similar list containing those orthologous to *Apis mellifera*, and then find the intersection of the two lists. This is a very simple three-step analysis, but provides data that can otherwise be difficult to create. Similarly, in the case of orthologue analysis, lists are of considerable utility: to find all of the *D. melanogaster *genes that have orthologues in *A. gambiae *and in *A. mellifera *one could create a list of orthologues between *D. melanogaster *and *A. gambiae *and a list of orthologues between *D. melanogaster *and *A. mellifera *and then find the intersection of the two lists. In general, the provision of lists means that more complicated queries can be built up in stages, with the output at each stage available for close examination, validation and manual pruning.

Lists also have an application in the comparison of entire data sources. The benefit of combining data sources through their union or intersection depends on the nature of the two datasets being combined. Different datasets, which have a low false positive but high false negative rate, can be combined via a union to increase the overall coverage of positives. Alternatively, for datasets that have high false positive and low false negative rates, analysis of their intersection may enrich for the most reliable data - that is, the subset of the two datasets most likely to be true positives [2]. Each 'aspect' allows easy access to all the data from a particular data source, making creation of specific lists and their comparison a straightforward task.

### Future directions

FlyMine is still in a phase of rapid development. The system is engineered to accommodate additional types of data and we aim to add new functional genomics data sources and increase coverage to further insects as well as other model organisms as data become available. The inclusion of data from other *Drosophila *species and other insects (for example, the silkworm, *Bombyx mori*) will allow interesting cross-species comparisons to be performed. Apart from the two already covered by FlyMine, ten other *Drosophila *species have now been sequenced and assembled [61]: we will load annotation and comparative genomics data for these along with tools to aid in their analysis and visualization. We also plan to add many small data sets so they may be queried and viewed in the context of existing large scale data.

As well as further development of the web interface, further data sources, templates and tools will be added. In the latter case we are interested in adding both tools to improve data visualization as well as those to allow data mining, and input from user communities is welcomed. A current focus of activity is on increasing the functionality available through list details pages: further viewing and analysis tools will be added. For example, for a list of genes, we will provide a visual representation of the chromosomal locations of the genes and provide information on potential commonalities of genes within the list by looking for statistically enriched use of GO terms within the set. We will increase the number of graphical summaries of object sets, and, as for the FlyAtlas data summary graph, will allow lists containing further subsets of the data to be generated by clicking on different columns of the graph. In addition to tools available on list details pages, other tools will be included, such as graphical viewing of interaction data with the ability to overlay other data sets (for example, expression data and GO annotations). While it will not always make sense to integrate tools closely with FlyMine, it is easy to generate different tabular data formats, and we aim to make it as easy as possible to export data for use in other applications, or to render sets of objects (for example, genes) through other web resources such as KEGG [62] and Reactome [63]. In this way we hope the utility of FlyMine to browse, query, analyze and visualize diverse integrated datasets will increase. To increase access between FlyMine and other resources we also plan to add support for querying FlyMine via web services.

### Availability, contact and help

The FlyMine query interface can be accessed from the FlyMine website [64]. From here there is access to help in the form of tutorials and a user manual. The help pages are under continued development. In addition, a feedback form is available from query pages that can be used to ask for help with queries or to provide us with comments or suggestions. This feedback form will automatically send us the query that is currently being worked on, making it easier to give an accurate response. The FlyMine team includes biologists experienced in using the web interface who will respond to help requests from users and add new templates as required. Further information is available by joining one of the FlyMine electronic mailing lists (details on the website) or by email to info@flymine.org. Comments and suggestions for improvements, new functionality and additional data sources are welcome.

## Conclusion

FlyMine is a new source of integrated data that allows researchers to make use of the huge amounts of high-throughput data currently being generated. The above examples provide a few illustrations of the way data can be manipulated in FlyMine. The number of possible combinations of data is large and will continue to grow and become more comprehensive as new and different types of data are added. The structure of FlyMine means researchers can rapidly accumulate a wealth of information about a particular object or set of objects, facilitating the formulation of new hypotheses for refining subsequent investigations. In addition to refinement and extension of smaller scale investigations, FlyMine can also facilitate whole genome approaches by allowing the investigation of networks and interactions among genome-wide datasets. The addition of graphical viewing and analysis tools and further data export options will greatly improve the ability to analyze data at this level.
